# *In vivo *MRI-based simulation of fatigue process: a possible trigger for human carotid atherosclerotic plaque rupture

**DOI:** 10.1186/1475-925X-12-36

**Published:** 2013-04-23

**Authors:** Yuan Huang, Zhongzhao Teng, Umar Sadat, Jing He, Martin J Graves, Jonathan H Gillard

**Affiliations:** 1 University Department of Radiology, University of Cambridge, Level 5, Box 218, Hills Road, Addenbrooke’s Hospital, Cambridge, CB2 0QQ, UK; 2 Department of Engineering, University of Cambridge, Cambridge, UK; 3 Cambridge Vascular Unit, Addenbrooke’s Hospital, Cambridge, UK

**Keywords:** Atherosclerosis, Carotid, MRI, Fatigue, Rupture

## Abstract

**Background:**

Atherosclerotic plaque is subjected to a repetitive deformation due to arterial pulsatility during each cardiac cycle and damage may be accumulated over a time period causing fibrous cap (FC) fatigue, which may ultimately lead to rupture. In this study, we investigate the fatigue process in human carotid plaques using *in vivo* carotid magnetic resonance (MR) imaging.

**Method:**

Twenty seven patients with atherosclerotic carotid artery disease were included in this study. Multi-sequence, high-resolution MR imaging was performed to depict the plaque structure. Twenty patients were found with ruptured FC or ulceration and 7 without. Modified Paris law was used to govern crack propagation and the propagation direction was perpendicular to the maximum principal stress at the element node located at the vulnerable site.

**Results:**

The predicted crack initiations from 20 patients with FC defect all matched with the locations of the *in vivo* observed FC defect. Crack length increased rapidly with numerical steps. The natural logarithm of fatigue life decreased linearly with the local FC thickness (R^2^ = 0.67). Plaques (n=7) without FC defect had a longer fatigue life compared with those with FC defect (p = 0.03).

**Conclusion:**

Fatigue process seems to explain the development of cracks in FC, which ultimately lead to plaque rupture.

## Introduction

Rupture of atherosclerotic plaque with resulting thromboembolism is a predominant cause of ischemic events such as myocardial infarction and cerebrovascular accidents [1]. Traditionally, luminal stenosis has been used to assess the severity of atherosclerosis. However, angiography identifies only those lesions encroaching significantly upon the lumen, and there is a poor correlation between the angiographic appearance and subsequent risk of plaque rupture, e.g. more than 80% of rupture events occur in lesions that cause less than 70% luminal stenosis in coronaries [2]. There is, therefore, a need for a diagnostic technique that provides comprehensive information about plaque morphology, function and structural stability in addition to luminal stenosis.

It has been widely hypothesized that plaque rupture occurs when the external loading due to blood pressure and flow exceeds the intrinsic fibrous cap strength [3-5]. With this relevance, *in vivo* image-based mechanical analysis has been employed to refine patient stratification [6-8] and predict subsequent symptom [9,10]. This hypothesis implies that rupture will not occur as long as stresses are below the strength of the cap. The experimental studies in animals and human subjects have reported pressures needed to cause plaque rupture being 2–10 times higher than the maximum pressure which clinically resulted in sudden plaque rupture [11,12]. However, it is known from the mechanical point of view that material (such as plaque) exposed to repetitive deformations (such as cardiac cycles in case of arteries) undergoes a fatigue process which leads to the final step of rupture. Fatigue is an incremental failure progression under influence of repetitive stresses, which results in acute failure at pressure levels seemingly much lower than the material tear strength [13]. Moreover, patients are frequently exposed to potential triggers without an inevitable acute morbid event [14], showing the limitation of modeling the plaque rupture as a single-step process. It seems that plaque rupture is not well characterized by considerations based on nominal strength or critical stress alone. The repetitive deformations caused by the cardiac cycle would therefore be another important factor affecting plaque stability, as compellingly proposed by Born [15] and Bank et al. [16].

McCord evaluated the effect of mechanical tension in arteries by observing morphological and mechanical changes caused by cyclic fatigue on diseased arteries in the areas of maximum stress [17]. Microscopic analysis demonstrated structural damage in the fatigued specimens as compared to normal tissues. Gilpin et al. observed cracks in porcine coronary arteries when fatigue test was performed [18]. Although efforts have been made to explore the fatigue process in the living tissues [13,17,18], little investigation has been conducted to apply the concept in the numerical study of human carotid atherosclerotic plaques. In this study, we explore the fatigue process in human carotid artery plaques using *in vivo* high-resolution MR imaging by assessing the propagation of cracks leading to plaque rupture.

## Materials and methods

(1) MRI acquisition

**Figure 1:**
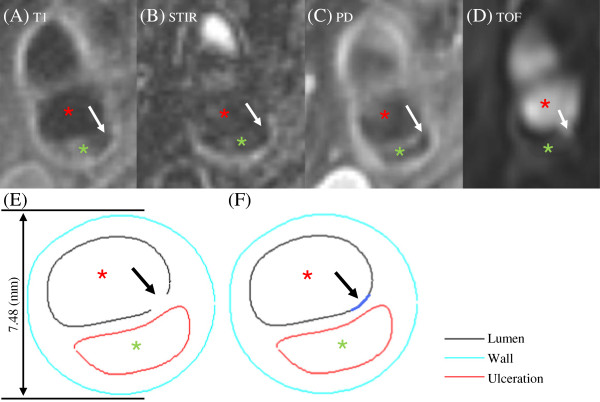
**High resolution, multi-sequence MR image and corresponding segmentation contour. **(**A**-**D**): MR images showing fibrous cap rupture (marked by white arrow) and ulceration; (**E**): segmented contour with a defect lumen due to fibrous cap rupture; and (**F**): cubic spline function was used to bridge the gap (thick blue line) to recover the intact lumen. Red asterisk: lumen; Green asterisk: ulceration.

Twenty seven patients (Table 1) with atherosclerotic carotid artery disease were included in this study. The study protocol was reviewed and approved by the Cambridgeshire Research Ethics Committee and written informed consent was given. Patients underwent high-resolution electrocardiograph (ECG)-gated black-blood MR imaging in a 1.5T MR system (Signa HDx GE Healthcare, Waukesha, WI) within 72 hours of the onset of symptom. After an initial coronal localizer sequence, axial 2D time-of-flight (TOF) MR angiography was performed to identify the location of the carotid bifurcation and the region of maximum stenosis. The following MRI protocol was used to delineate various atherosclerotic components (Figure 1A-D), such as fibrous cap (FC), lipid-rich necrotic core (LRNC) and plaque hemorrhage (PH): T_1_ weighted (repetition time/echo time: 1*RR/7.8 ms) with fat saturation; T_2_ weighted (repetition time/echo time: 2*RR/100 ms) with fat saturation; and STIR (repetition time/echo time/inversion time: 2*RR/46 ms/150 ms) [19]. The in-plane spatial resolution achieved was 0.39 × 0.39 mm^2^ and the slide interval was 3 mm. The total scan duration was around 45 minutes. Manual segmentation of plaque components (Figure 1E & F) was performed by two MR readers (US and JHG) in agreement based on net signal intensities from different sequences using previously published criteria [8], to identify FC, LRNC, calcification (Ca) and PH in CMRTools (London, UK). Compositional features depicted by MR have been validated using histological data [20]. FC defects (rupture or ulceration) were found in 20 patients.

(2) Fatigue simulation

In the patient group with FC defect, for each plaque one MR slice with presence of FC rupture or ulceration (Figure 1) was used for the fatigue simulation and the ulceration was replaced by LRNC with the assumption that the ulceration was the result of escaped LRNC [21-23]. As the intact lumen contour was no longer available for slices with ruptured FC (Figure 1E), cubic spline function was used to bridge the gap to recover the intact FC configuration (Figure 1F). MR images were acquired during diastole and arterial lumen remained pressurized. In order to achieve a zero-pressure configuration as the start shape for the numerical simulation, a non-uniform shrinkage procedure [24] was employed. Briefly, a uniform shrinking procedure [25] was applied to compensate the pressurization followed by a non-uniform refinement [24] to restore lumen irregularity. The shrinkage rate was determined by the best match between the predicted pressurized lumen contour and the one obtained from *in vivo* MR image. A cross-sectional area was modeled representing the plane strain state, which allowed a 2D analysis to be performed. The stress was expressed using Von Mises’ criterion [26]. The modified Paris-relation was used to describe the crack propagation [13,27]:

(1) $$\frac{\mathrm{dl}}{\mathrm{dn}}=C{\left(\sigma_{\text{max}}-\sigma_{\text{min}}\right)}^{\alpha }\sigma_{\text{max}}^{\beta }$$

where *l* is the crack length; *n* is the number of fatigue cycles defined by the heart beat; *C*, *α* and *β* are material constants. The fracture mechanism is not fully understood and the constants governing the crack propagation are not available, the conservative values were, therefore, used in this study [13]: *C* = 10^-16^ mm · kPa^-3^, α = 1 and *β* = 2. *σ*_*max*_ and *σ*_*min*_ are the maximum and minimum stresses during one cardiac cycle, respectively. The modified Paris-relation quantifies the crack propagation rate using a power law of pressure measures, with both the maximum value and dynamic variation being considered.

Per numerical step a crack increment governed by Eq.(1) was applied at the element node at the crack tip, which was determined by the previous numerical step and the incremental direction was perpendicular to the maximum principal stress. That was with the following assumptions [28]: (a) In this study, the maximum principal stress was much bigger than those in other principal directions. The fatigue crack was, therefore, assumed to be under model-I loading; (b) For the pure model-I, the pre-crack would orient along the maximum principal direction; and (c) The further fracture would orient in the same direction as the pre-crack in order to maximize the energy release rate according to the Griffith theory or the maximum energy release rate theory. The crack increment was, therefore, along the direction perpendicular to the maximum principal stress. However, this direction would change after each numerical step according to local stress conditions. The numerical simulation stopped when the crack reached the enclosed atherosclerotic component, and the fatigue cycles, *n*, were calculated [13]:

(2) $$n=\overset{N}{\underset{i=1}{\sum }}\frac{l_{i}-l_{i-1}}{{\left(\frac{\mathrm{dl}}{\mathrm{dn}}\right)}_{i}}$$

in which *N* is the numerical steps. The fatigue life was defined as the fatigue cycles when the crack reached the component.

The failure initiation was not purposely predefined according to the MR images from which the FC rupture was observed. It was assumed to be identical with the location of high stress concentration over the diseased region, where the lumen curvature was locally large or the FC thickness was locally small [29]. This assumption was based on the fact that plaque rupture often occurred at the shoulder area with large lumen curvatures, and the location with minimum FC thickness, where the material was focally weak [3]. The simulation was, therefore, firstly performed with an intact FC; the initial crack was then generated over the FC using this criterion; and further simulation was performed to drive the crack propagation governed by Eq.(1). Following this procedure, the agreement between the simulated failure initiation and propagation and the location of *in vivo* ulceration and FC rupture was quantified.

Fatigue simulation was also performed with the MR slice at the most stenotic site from patients (7 in total) without any MR-depicted FC defect. The failure initiation was assumed using the above criterion and the simulated fatigue life was compared with those with MR-depicted FC defects.

**Figure 2:**
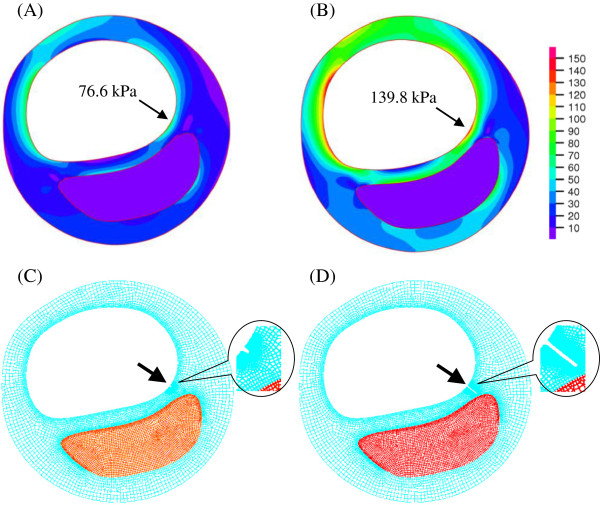
**Band plot of Von Mises stress and fatigue initiation and crack propagation. **(**A** &**B**): stress band plots at diastole and systole, respectively; (**C**): fatigue initiation at the vulnerable site (marked by arrow); and (**D**) crack propagation at the 7th numerical step.

All plaque components including fibrous tissue, lipid core, calcification and healthy arterial wall were assumed to be non-linear, isotropic and hyper-elastic. The modified Mooney-Rivlin strain energy density function was used to describe the material [30]:

(3) $$W=c_{1}\left(I_{1}-3\right)+D_{1}\left[\text{exp}\left(D_{2}\left(I_{1}-3\right)\right)-1\right],\;I_{1}=C_{\mathrm{ii}}$$

where *I*_1_ is the first deformation invariant and *C* is the right Cauchy-Green deformation tensor. Material- parameters *c*_1_ and *D*_*i*_ (*i* = 1, 2) were chosen to match available experimental measurement data [4,31-33]: vessel material, *c*_1_ = 36.8 kPa, *D*_1_ = 14.4 kPa, *D*_2_ = 2; fibrous cap, *c*_1_ = 73.6 kPa, *D*_1_ = 28.8 kPa, *D*_2_ = 2.5; lipid-rich necrotic core, *c*_1_ = 2 kPa, *D*_1_ = 2 kPa, *D*_2_ = 1.5; calcification, *c*_1_ = 368 kPa, *D*_1_ = 144 kPa, *D*_2_ = 2.0. A pulsating pressure calibrated using the systolic/diastolic arm pressure value of each patient at the time of the MR imaging was imposed in the lumen when numerical simulation was performed. Pressure at the out-boundary of each vessel slice was set to zero. A much finer mesh was created around the crack region to improve the accuracy of simulation. The stress was calculated by ADINA8.6.1 (Adina, R&D, MA, USA) as shown in Figure 2A & B.

(3) Statistical analysis

For the data set not passing the normality test (Shapiro-Wilk test), the two-tailed Mann–Whitney test was used for the statistical analysis and the data would be presented in median [inter quartile range] (IQR); otherwise, the two-tailed student *t* test was used and the data would be presented in mean ± standard deviation (SD). Two-sided Fisher’s exact test was used to analyze the difference in contingency tables. The statistical analysis was performed in Instat3.06 (GraphPad Software Inc., CA, USA). A significant difference was assumed if p-value < 0.05.

**Table 1:** Patient demography (n = 27)

**Total number of patients/value**	**With FC defect (n = 20)**	**Without FC defect (n = 7)**	**p-value**
Age (year)	76.2 ± 10.3	71.3 ± 21.3	0.88
Female, n (%)	8 (40.0)	2 (28.6)	0.68
Systolic Pressure (mmHg; Mean ± SD)	146.0 ± 20.1	127.7 ± 27.9	0.09
Diastolic pressure (mmHg; Mean ± SD)	80.7 ± 14.3	72.3 ± 15.8	0.23
Heart rate (/minute) [IQR]	72 [72, 74]	72 [72, 72]	0.71
Hypertension, n (%)	10 (50.0)	4 (57.1)	1.00
Diabetes, n (%)	3 (15.0)	0 (0)	1.00
Renal impairment, n (%)	1 (5.0)	1 (14.3)	0.46
Ischemic heart disease, n (%)	8 (40.0)	1 (14.3)	0.36
Peripheral vascular disease, n (%)	2 (10.0)	1 (14.3)	1.00
Coronary artery disease, n (%)	0 (0)	0 (0)	1.00
Previous TIA/Stroke, n (%)	7 (35.0)	1 (14.3)	0.63
ECST defined Stenosis (%; Mean ± SD)	56.4 ± 14.9	60.5 ± 21.7	0.60

*IQR*: inter quartile range.

## Results

**Figure 3:**
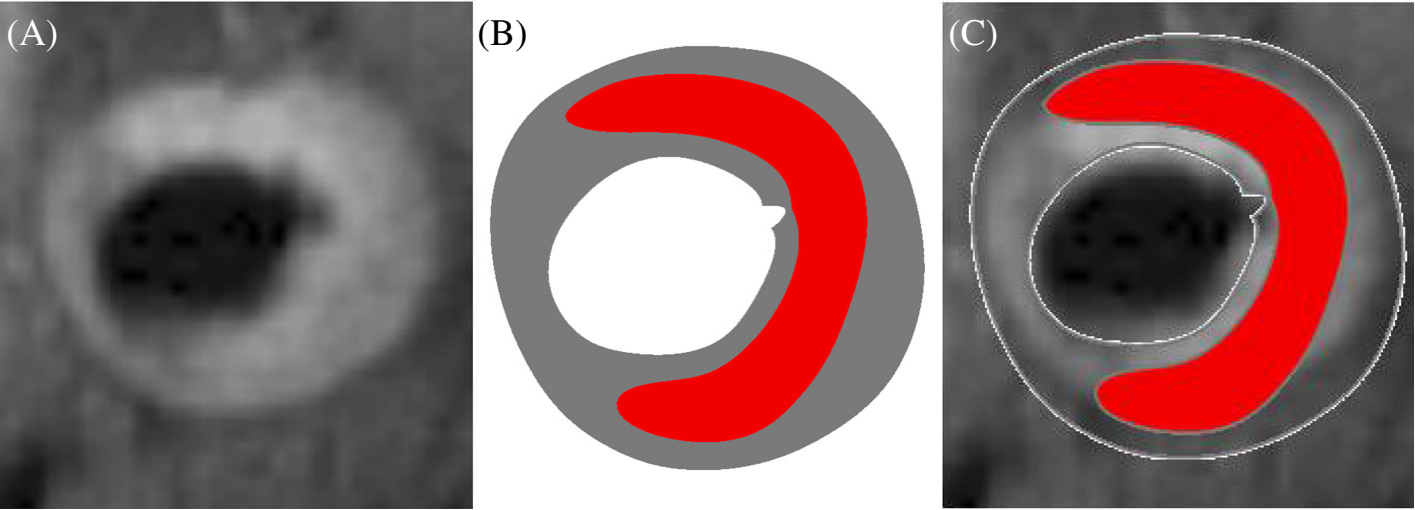
**The agreement between the location of crack and *in vivo *ulceration. **(**A**): *in vivo *MR image showing plaque ulceration; (**B**) simulated plaque configuration with crack; (**C**) overlapped images in A and B showing the agreement between the location of crack and ulceration.

In total 27 MR slices (one from each patient) were used in this study, 20 with FC rupture or ulceration and 7 without any FC defect. According to the foregoing fatigue initiation criterion, an initial crack was generated after the first numerical step at the vulnerable site where the local maximum stress concentration at systole was found [29]. As shown in Figure 2C, a small initial crack (marked by arrow) was generated after the first numerical step at the site where local stress concentration was found (marked by arrow in Figure 2B). The crack propagated in the following numerical steps as shown in Figure 2D. By comparing with the corresponding *in vivo* MR images (Figure 1A-D), it was found that the simulated crack location and path were in a good agreement with the location of plaque ulceration (marked by white arrows in Figure 1A-D). In this study, all predicted fatigue cracks agreed with the location where FC rupture/ulceration was observed in the *in vivo* MR image. Seventeen out of 20 (85%) slices had the cracks propagate radially and eventually reach the enclosed atherosclerotic component. The location agreement between the predicted crack and *in vivo* FC rupture and ulceration was assessed by checking their positions after the lumen contours being best matched (Figure 3).

**Figure 4:**
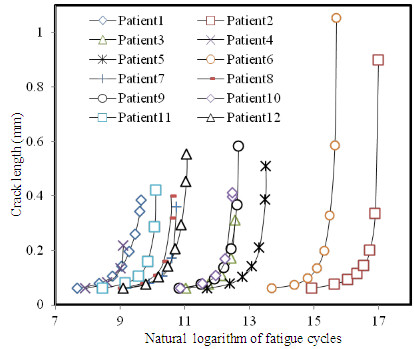
Crack length increased rapidly with fatigue cycles (the first data point of each patient was removed for clearness as it was 0 in the abscissa axis; for clearness, results from randomly selected 12 patients were shown).

**Figure 5:**
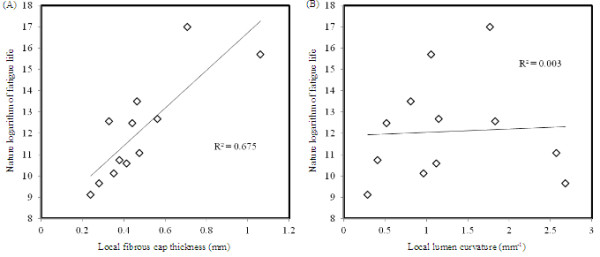
**The relationship between the fatigue life, local fibrous cap thickness and lumen curvature. (A)**: fatigue life increased with the local fibrous cap thickness; **(B)**: fatigue life did not associate with local lumen curvature.

Further investigation showed that the crack length increased rapidly with numerical steps and it had an exponential relationship with the natural logarithm of the fatigue cycles as shown in Figure 4. This indicated that once the fatigue crack was initiated, it developed exponentially during further loading cycles. Moreover, a plaque with a thin fibrous cap would have a much shorter fatigue life. As shown in Figure 5A, the natural logarithm of fatigue life decreased linearly with the local FC thickness (R^2^ = 0.675). Although when the FC is intact, stress has a close relationship with the local lumen curvature [34], as shown in Figure 5B, natural logarithm of fatigue life did not have any relationship with the local lumen curvature (R^2^ = 0.003).

**Figure 6:**
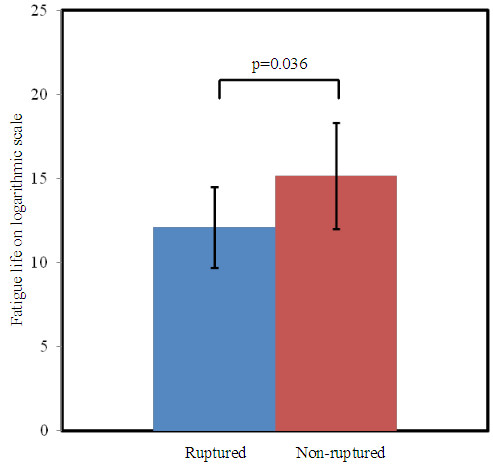
Comparison of fatigue life for ruptured and non-ruptured cases.

Although it is impossible to observe the *in vivo* crack propagation with current imaging technique, comparing the fatigue life of plaques with and without FC rupture or ulceration might provide indirect support for this hypothesis. With this consideration, the fatigue simulation was performed with the MR slice at the most stenotic site of 7 randomly-chosen patients with no shown rupture. The result indicated that the non-ruptured group was found to have a significantly longer fatigue life (Figure 6. ruptured: 12.23 ± 1.96, n = 20; without rupture: 15.13 ± 3.05, n = 7; p = 0.014. Fatigue life is shown on the natural logarithmic scale).

## Discussion

To the best of our knowledge, this is the first study to explore fatigue process in atherosclerotic plaques using *in vivo* MR imaging-based patient specific simulations. We found that fatigue cracks initiated at the vulnerable sites which ultimately had developed FC rupture. The direction of crack propagation was radial and the crack ultimately reached the underlying plaque component. Moreover, once the fatigue crack was initiated, it developed with an exponentially increasing rate during further loading cycles. These results are in accordance with the first report by Versluis et al. using idealized models [13]. In addition, based on the fact that plaque stability can be characterized by assessing fibrous cap thickness and lumen curvature, we also explored the relation between these parameters and the fatigue life.

The concept of fatigue process is in contrast with our common perception that high structural stresses result in plaque rupture. Fatigue tends to occur at stresses much lower than the tissue -tear strength. Although it is a quiescent process and is not associated with any symptomology as compared to plaque rupture, its importance cannot be overlooked. It has been reported that the ultimate material strength of an intact FC of carotid plaque is about 1000 kPa [35], while the critical mechanics stress in the ruptured plaque is about 500 kPa [5,36]. Therefore, peaks in blood pressure alone may not be sufficient to cause a plaque to fissure. Plaque rupture may be an insidious accumulation of damage induced by cyclic stresses. As a general physical process, fatigue can happen in any material when it subjects to a periodic loading. Being an active biological structure, FC interacts with the local mechanical environment, such as stress distribution, and adapts accordingly. In the normal physiological conditions, living components within the FC structure appear to be dormant and synthesis and degradation of connective tissues are balanced. However as an injury, the fatigue crack may attract inflammatory cells, such as macrophage [37,38]. These inflammatory cells secrete cytokines and proteolytic enzymes into the extracellular matrix, resulting in both decreased synthesis and an enhanced destruction of extracellular matrix [39]. Diminished collagen synthesis will weaken the fibrous cap [40], and therefore lead to a greater tendency to rupture at lower stress levels by repeated cyclic loading.

Besides the local tissue structural strength, other factors which would affect the fatigue life are the heart rate, mean blood pressure and pulse pressure as it could be seen from Eq.(1) [13]. These were not explored in this patient-specific study but have been exquisitely investigated by Ku’s group [17,18] and Versluis et al. [13]. Decreased heart rate, arterial pressure and pulse pressure will prolong fatigue life by decreasing the number of stress cycles or the magnitude of the low-level cyclic stress [16]. Modulation of these factors by appropriate medication will affect the fatigue life of a plaque, such as beta blockers by lowering the heart rate. Anti-hypertension medication will decrease blood pressure and therefore decrease the stress concentration level and eventually increase the fatigue life. Use of cholesterol lowering agents such a statins by reducing the atherosclerosis-related inflammation will enhance the plaque stability and thus also prolong the fatigue life. There is already plenty of clinical evidence to support that uncontrolled blood pressure [41] and high heart rate [42,43] is associated with poor cardiovascular outcomes. This study provides an insight into underlying mechanics of this interesting phenomenon. The numerical results also suggest the importance of best medical therapy which is already used for patients with atherosclerotic plaque disease i.e. use of anti-hypertensives, cholesterol lowering agents and anticoagulants.

But can we assess the fatigue life of a tissue to predict when plaque rupture will occur? The answer is no. This is because there is no investigative technique at present which can provide us with the real time information about the plaque material strength, critical mechanical conditions and presence of fatigue crack. Furthermore, the blood pressure (mean and pulse) and heart rate undergo variations in day-to-day life. In addition, other factors like dietary habits, emotional status, other co-morbidities and intake of various medications also affect the above. Therefore, strictly speaking, such an estimation of fatigue life is inaccurate and even unrealistic. To add to this limitation, the first step of fatigue process i.e. nucleation (could be characterized by the endothelial injury in arteries) cannot be identified when it occurs, as it is asymptomatic.

Although the crack growth governed by Eq.(1) could predict FC rupture as shown in this study, Eq.(1) may over-simplify the fatigue mechanism of atherosclerotic plaque. Compared with metal whose fatigue behavior has been widely quantified, plaque is a more complex structure with various atherosclerotic components and anisotropic material properties. Experimental investigation is, therefore, needed to have a better understanding of the plaque failure mechanism. Moreover, as the fatigue life reflects the structural resistance to fatigue-type failure, this suggests that plaques without ulceration are much less prone to rupture in the presence of micro-fissures. It is also worth noting that due to the currently limited data on material properties, no fatigue threshold was incorporated here. Since previous studies have shown the ulcerated models to predict much higher structural stress than others [5], the introduction of the threshold would make the crack in the non-ruptured group not only less likely to initiate but also much slower in terms of propagation.

Instead of employing a fracture line and the concept of J-integral, a blunt crack was incorporated into the computational model based on several considerations. Firstly and most importantly, based on the general behavior of soft living tissues and its biological environment, a sharp fracture line is unlikely to be observed. Secondly, crack blunting could be an important feature in the propagation of cracks. This has been investigated in a number of experimental, theoretical and numerical studies [30,44-46]; some proposed that crack tip blunting and folding could be the underlying mechanism of crack propagation. Lastly, as J-integral is based on the theory of deformation plasticity it could not be applied to cases with unloading. This makes the J-integral approach inappropriate for the simulations of fatigue. Despite efforts have been made on the extension of the concept (such as the cyclic J-integral) [47,48], there remains some ambiguity in defining the proper limits of integration [28]. Moreover, this cyclic J approach may severely violate the basic assumptions leading to the development of the J-integral [28].

This study has limitations such as: (1) the FC thickness measurement was critical in this study. However, due to MR spatial resolution limited to 0.39 mm, there is a possibility that thinner FCs below this resolution have been underestimated; (2) the inter-observer difference in FC thickness and the size of atherosclerotic component might cause a big difference in numerical result [49]. In this study, the MR images were segmented by two MR readers in agreement with each other; (3) it was a 2D structure-only simulation with the effect from blood flow ignored; (4) the residual stress within the plaque was not considered since it is not measurable with current MR technique [50]; (5) the plaque was treated as a piecewise homogenous, isotropic material and the patient-specific material properties were not considered; and (6) the crack initiated at any loading level and no threshold was considered.

In conclusion, results obtained in this study shed light on a different mechanism of FC rupture-- fatigue. Further experimental study is needed to validate its existence.

## Competing interest

The authors declare that they have no competing interests.

## Authors’ contribution

YH and JH were responsible for performing the mechanical simulation and results analysis; ZT designed the concept, analyzed the data and wrote the manuscript; US recruited the patient, collected and analyzed the raw data and revised the manuscript; MJG developed the MR sequences and JHG revised the manuscript. All authors read and approved the final manuscript.
